# Pyruvate Regulates the Expression of *DLAT* to Promote Follicular Growth

**DOI:** 10.3390/cells14060444

**Published:** 2025-03-17

**Authors:** Liuhong Zhang, Yixuan Guo, Enyuan Huang, Jianing Lu, Tiantian Wang, Yonghua Shi, Meng Lv, Yongcai Chen, Shuo Li, Xiaolong Yuan, Jiaqi Li

**Affiliations:** 1 State Key Laboratory of Swine and Poultry Breeding Industry, National Engineering Research Center for Breeding Swine Industry, Guangdong Provincial Key Laboratory of Agro-Animal Genomics and Molecular Breeding, College of Animal Science, South China Agricultural University, Guangzhou 510642, China; zhanglh@stu.scau.edu.cn (L.Z.); gyx051129@stu.scau.edu.cn (Y.G.); heeeyy@stu.scau.edu.cn (E.H.); ljn@stu.scau.edu.cn (J.L.); wtt1018@stu.scau.edu.cn (T.W.); z20223540@stu.sxau.edu.cn (Y.S.); lvmeng@sysucc.org.cn (M.L.); cyc0915@stu.scau.edu.cn (Y.C.); ls4000@stu.scau.edu.cn (S.L.); 2 Shenzhen Branch, Guangdong Laboratory for Lingnan Modern Agriculture, Genome Analysis Laboratory of the Ministry of Agriculture, Agricultural Genomics Institute at Shenzhen, Chinese Academy of Agricultural Sciences, Shenzhen 518120, China; 3 College of Animal Science, Shanxi Agricultural University, Jinzhong 030801,China; 4 Centre for Healthy Ageing, Health Futures Institute, Murdoch University, Murdoch, WA 6150, Australia

**Keywords:** follicular growth, granulosa cells, pyruvate, *DLAT*

## Abstract

Increasing evidence has suggested that dihydrolipoamide S-acetyltransferase (*DLAT*), a subunit of the pyruvate dehydrogenase complex, is crucial for pyruvate metabolism and the regulation of cell death. The excessive death of granulosa cells (GCs) hinders the progression of follicular growth. However, the relationship between *DLAT* and follicular growth is poorly understood. Here, we found that pyruvate significantly shortened the age of pubertal initiation in mice and promoted follicular growth by promoting the proliferation of GCs. In addition, pyruvate up-regulated the expression of *DLAT* and the high level of *DLAT* was observed in large follicles, which were associated with follicular growth. Mechanistically, *DLAT* increased the mRNA and protein levels of proliferation pathways such as *PCNA* and *MCL1* to promote GC proliferation. Additionally, DLAT bound to CASP3 and CASP9 proteins to inhibit the apoptosis of GCs. Taken together, these results reveal a mechanism that pyruvate regulated *DLAT* to promote follicular growth, and *DLAT* represents a promising target that supports new strategies for improving the growth of follicles.

## 1. Introduction

The follicle serves as the fundamental functional unit of the ovary, and only 1% of follicles undergo ovulation, and the remainder undergo atresia [1,2]. Granulosa cells (GCs) play an important role in follicular growth [3,4]. Specifically, the proliferation of GCs drives primordial follicle growth to preantral follicles, antral follicles, and mature follicles [5] and promotes oocytes maturation and ovulation [6,7,8]. Conversely, excessive apoptosis of GCs inhibits follicular growth [9] and leads to considerable follicular atresia in mice [10,11]. Furthermore, studies have found that the count of antral follicles is positively correlated with female fertility [12,13]. For instance, cows with a lower number of antral follicles exhibit smaller ovaries and lower pregnancy rates compared to those with a higher number of antral follicles [13]. Thus, sustaining healthy proliferation of GCs is fundamental to promote follicular growth and improve female fertility.

Previous studies have shown that pyruvate serves as a key metabolic substrate and provides energy for follicular growth [14,15]. As a precursor for the tricarboxylic acid (TCA) cycle, pyruvate is oxidized to acetyl-CoA via pyruvate dehydrogenase complex and then enters the TCA cycle to generate ATP for normal cellular activities [16,17,18]. Pyruvate deficiency blocks embryo growth at the two-cell stage and leads to pregnancy failure [19,20]. Pyruvate supplementation increases the level of anti-apoptotic protein BCL2 and inhibits the senescence of mouse oocytes [21]. Additionally, pyruvate reduces reactive oxygen species levels and increases glutathione levels, thereby promoting follicular growth to ovulation [22,23,24,25]. However, how pyruvate regulates GCs and follicular development remains unclear.

Dihydrolipoamide S-acetyltransferase (*DLAT*), a vital enzyme in the metabolic pathway of pyruvate, is involved in regulating the cellular energy supply [26,27]. It catalyzes the transfer of acetyl groups from dihydrolipids to coenzyme A (CoA) and converts pyruvate to acetyl-CoA for the TCA cycle, which is crucial for cellular energy generation [17,28]. The current study suggests that *DLAT* contributes significantly to the development of multiple diseases [29]. For example, silencing *DLAT* inhibits gastric cancer cell proliferation and migration [30], while *DLAT* overexpression promotes liver hepatocellular carcinoma cell growth by targeting autophagy [31]. However, studies on the regulations of *DLAT* in follicular growth are still limited.

Our findings indicated that pyruvate promoted GC proliferation and up-regulated the expression of *DLAT.* Therefore, we hypothesized that pyruvate might regulate GC proliferation and apoptosis by altering *DLAT* expression levels, thereby promoting follicular growth and advancing the age of pubertal initiation in mammals. This study helps us to understand the role of *DLAT* in follicular development and provides new insights into potential biomarkers and therapeutic targets for improving follicular growth.

## 2. Materials and Methods

### 2.1. Mouse Experiments

Female C57BL/6 mice that were three weeks old were provided by the Guangdong Medical Laboratory Animal Center (Guangzhou, China). In the pyruvate treatment groups, the mice were randomized into 2 groups (pyruvate and blank) with 6 individuals in each group and were intraperitoneally injected once a day for three weeks. In the lentivirus treatment groups, the mice were randomized into 4 groups (LV-NC, LV-DLAT, sh-NC, and sh-DLAT) with 6 individuals in each group and were intraperitoneally injected once a week for three weeks. The estrus status of the mice was recorded daily, and the pubertal initiation of the mice was estimated by the opening of the vaginal orifice.

### 2.2. Cell Lines, Culture, and Treatment

Human ovarian granulosa cell lines (KGN, CL-0603) were purchased from Wuhan Priceline Biotechnology(Priceline Biotechnology, Wuhan, China).; were maintained in DMEM (HyClone, Logan, UT, USA) supplemented with 10% FBS (Gibco, Waltham, MA, USA), 1% penicillin (100 units/mL), and streptomycin (100 μg/mL) (Gibco, Waltham, MA, USA); and were incubated at 37 °C in a 5% CO_2_ atmosphere. Passaging occurred when cell confluence reached over 80%.

The cells were seeded and cultured into six-well plates. When the cells reached 80% confluence in each well, the OE-NC, OE-DLAT, si-NC, and si-DLATs (Ribo, Guangzhou, China) were transfected into cells by using LipofectamineTM 3000 Reagent (Invitrogen, Waltham, MA, USA) in antibiotic-free medium for 6 h. After 6 h, the medium was changed to fresh medium for the subsequent experiments.

### 2.3. Follicles Culture

The ovaries were sourced from a slaughterhouse located in Guangzhou, and the slaughter process complies with the Chinese slaughter execution standards. The 3–5 mm follicles were isolated from the ovaries of the sows using a scalpel blade and cultured in DMEM/F12 (HyClone, Logan, UT, USA) supplemented with 1% penicillin (100 units/mL) and streptomycin (100 μg/mL) (Gibco, Waltham, MA, USA) in 24-well plates. They were incubated at 38.5 °C in 5% CO_2_ for 24 h and then treated with lentivirus. Photographs of the follicles were taken on the first, third, and fifth days after treatments.

### 2.4. Hematoxylin and Eosin (H & E)

In this study, hematoxylin-eosin staining (Servicebio, Wuhan, China) was used to detect the ovulation of mice ovaries. Ovarian tissues were fixed with 4% paraformaldehyde, embedded in paraffin, and sectioned into maximum transverse slices. These sections were then stained with HE. The photographs were observed using an inverted microscope (Leica, Wetzlar, Germany) and the images were captured using a DS-Fi3 microscope camera (Nikon, Tokyo, Japan).

### 2.5. TUNEL Assay

In this study, a TUNEL assay kit (Servicebio, Wuhan, China) was used to evaluate GC apoptosis in the ovaries of mice, and the specific experimental procedures were conducted following the manufacturer’s guidelines. The TUNEL stained sections were observed using an inverted microscope (Leica, Wetzlar, Germany) and the images were captured using HCImage Live v4.x64 software (Iwata, Japan). Specifically, for DAPI staining, the excitation wavelength was 330–380 nm (UV) and the emission wavelength was 420 nm (blue); for the 488 fluorescein, the excitation wavelength was 465–495 nm, and the emission wavelength was 515–555 nm (green).

### 2.6. RNA and RT-qPCR

To analyze the mRNA levels, we extracted the total RNA from the samples using TRIzol reagent (Invitrogen, Waltham, MA, USA) following the manufacturer’s instructions. The RNA concentrations were measured spectrophotometrically with a NanoDrop One (NanoDrop, Thermo, Waltham, MA, USA). Subsequently, the RNA was reverse-transcribed into cDNA according to the instructions of the Primescript RT Master Mix (Takara, Japan). Real-time PCR was performed to quantify the mRNA levels using Hieff^®^ qPCR SYBR Green Master Mix (Yeasen, Shanghai, China), with GAPDH serving as the endogenous control. The reactions were carried out on a LightCycler Real-Time PCR system. The relative gene expression levels were calculated based on the 2^−△△Ct^ method. All the procedures were repeated in at least triplicate. The primer sequences used for mouse are listed in Table 1; for human, they are listed in Table 2; and those used for pigs are listed in Table 3.

### 2.7. Western Blot

The total protein was extracted from each sample using a total protein kit (BestBio, Shanghai, China). The protein concentrations were then measured with a BCA Protein Assay Kit (Biosharp, Xianyang, China). The samples were denatured by boiling with SDS at a 4:1 ratio for SDS-PAGE. The denatured proteins were then transferred to polyvinylidene difluoride (PVDF) membranes (PVDF, Maplewood, MN, USA). The PVDF membranes were blocked with 5% skimmed milk in TBST at 37 °C for 2 h and incubated with primary antibodies DLAT (Proteintech, 1:5000, Wuhan, China), PCNA (Affinity, 1:1000, Liyang, China), MCL1 (Affinity, 1:1000, Liyang, China), CASP3 (Affinity, 1:1000), CASP8 (Affinity, 1:1000), CASP9 (Affinity, 1:1000, Liyang, China), or α-Tubulin (HUABIO, 1:5000) in TBST at 4 °C overnight and then incubated with secondary antibodies (Santa Cruz, Dallas, TX, USA, 1:20,000) at 37 °C for 2 h. All the proteins were visualized using the BeyoECL Moon (Beyotime, Shanghai, China). The exposure time for imaging varied depending on the signal intensity but typically ranged from 10 s to 2 min. Specific exposure times were optimized for each sample to ensure clear and accurate detection of the target protein without overexposure. The quantification was conducted via densitometric analysis using Image J 1.x software (Bethesda, MD, USA). The protein expression levels were normalized to the internal control α-Tubulin.

### 2.8. Co-Immunoprecipitation (Co-IP)

The specific experimental procedures followed the rProtein A/G Magnetic IP/Co-IP Kit (ACE Biotechnology, Nanjing, China). The cells were lysed with 1× Lysis/Wash Buffer and rinsed with rProtein A/G Magpoly Beads to remove non-specific binding proteins, followed by immunoprecipitation with DLAT antibody for 2 h. Finally, the antibody–antigen complexes were collected by incubation for 4 h with rProtein A/G Magpoly Beads. The obtained samples could be used directly for Western blot or stored at −20 °C.

### 2.9. EdU Assay

For the cell proliferation assay, the EdU Apollo567 Kit (Ribo, Guangzhou, China) was used. After 24 h of incubation, the cells were labeled with EdU (20 µM) at 37 °C for 2 h. The cells were then fixed with 4% paraformaldehyde, permeabilized with 0.5% Triton X-100, stained with Apollo R567 and Hoechst 33342, and photographed using an inverted microscope (Leica, Wetzlar, Germany), and the images were captured using HCImage Live v4.x64 software (Iwata, Japan).

### 2.10. Flow Cytometry

For the cell apoptosis assay, an Apoptosis Detection Kit (KeyGEN BioTECH, Nanjing, China) was used. After 48 h of incubation, the cells were stained with annexin V-FITC and PI at room temperature in the dark for 10–20 min and then the fluorescence was detected using a Guava^®^ easyCyte™ HT Flow Cytometer (Millipore, Molsheim, French). Annexin V-FITC and PI fluorescence were analyzed using 488 nm excitation, with emissions at 525 nm (FITC) and 585 nm (PI). FlowJo v10 software (Ashland, OR, USA) quantified early/late apoptotic and dead cell populations.

### 2.11. Plasmids

The NCBI website was logged into to obtain the sequence information of the DLAT gene (NCBI accession number: NM_001931.4). The primers were designed using Primer Premier 5.0 software, incorporating BamHI and XbaI restriction sites at the 5′ and 3′ ends, and the sequences were submitted to Sangon Biotech for synthesis. PCR amplification was performed, and the PCR product as well as the pcDNA3.1 vector (Pronega, Madison, WI, USA) were digested with BamHI (Thermo, Waltham, MA, USA) and XbaI (Thermo, Waltham, MA, USA) at 37 °C for 2 h, followed by purification using a gel extraction kit (Magen, Guangzhou, China). The digested fragments were ligated using T4 DNA ligase (TaKaRa, Kyoto, Japan) at a molar ratio of 3:1 (insert:vector) at 16 °C for 6 h. The constructed DLAT overexpression vector was verified by restriction enzyme digestion and Sanger sequencing to confirm the correct insertion of the DLAT gene. The specific sequence information used is listed in Table 4. The cleavage sites are in bold.

### 2.12. Statistical Analyses

The data in this research were presented as the mean ± standard deviation (SD). The Shapiro–Wilk test was used to determine that the data were normally distributed. Statistical significance was assessed using Student’s t-test for comparisons between two groups and one-way ANOVA for comparisons involving more than two groups. For all the figures, * *p* < 0.05, ** *p* < 0.01, *** *p* < 0.001, and ns indicates not statistically significance. The groups labeled with the same letter (e.g., c and c) indicate no significant difference (*p* > 0.05), while the groups labeled with different letters (e.g., a and c) indicate a significant difference (*p* < 0.05).

## 3. Results

### 3.1. Pyruvate Promotes Follicular Growth in Mice

**Figure 1:**
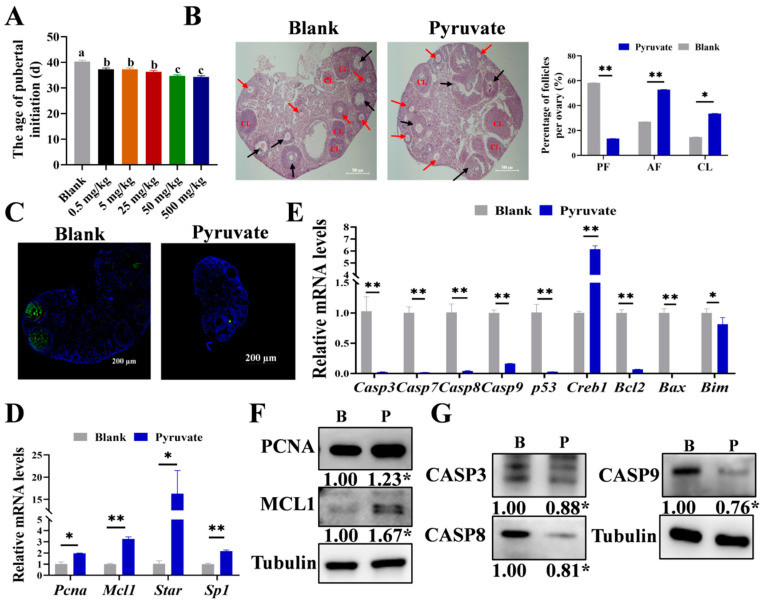
Pyruvate promotes follicular growth in mice. (**A**). The age of pubertal initiation of mice after treating with different concentrations of pyruvate. The groups labeled with the same letter (e.g., c and c) indicate no significant difference (*p* > 0.05), while the groups labeled with different letters (e.g., a and c) indicate a significant difference (*p* < 0.05). (**B**). The HE staining results of ovarian sections in the blank group and the pyruvate group. Black arrows: preantral follicle (PF), red arrows: antral follicle (AF), and CL: the corpus luteum. Scale bar: 500 μm (**C**). The TUNEL results of mouse ovarian sections, and the green fluorescence represents apoptotic cells. Scale bar: 200 μm. The mRNA levels of the proliferation signaling pathway (**D**) and apoptotic signaling pathway (**E**) in the ovaries of mice. The protein levels of the proliferation signaling pathway (**F**) and apoptotic signaling pathway (**G**) in mice ovaries: the blank group (B) and the pyruvate group (P). * *p* < 0.05; ** *p* < 0.01.

To examine the role of pyruvate in regulating follicular growth, five concentrations (0.5 mg/kg, 5 mg/kg, 25 mg/kg, 50 mg/kg, and 500 mg/kg) were set up for intraperitoneal injection into three-week-old mice, and it was found that the age of pubertal initiation in mice was notably shortened in a dose-dependent manner after pyruvate treatment (Figure 1A), and a concentration of 50 mg/kg was used for the subsequent experiments. After pyruvate treatment, the proportions of antral follicles (AFs) and corpus luteum (CL) significantly doubled, while the proportion of preantral follicles (PFs) significantly decreased from 58% to 13% (Figure 1B). The apoptosis of GCs in the ovaries significantly declined after pyruvate treatment (Figure 1C). Pyruvate significantly elevated the mRNA expression levels of *Pcna*, *Mcl1*, *Star*, and *Sp1*, as well as the protein levels of PCNA and MCL1 (Figure 1D,F). In addition, pyruvate significantly reduced the mRNA and protein levels of *CASP3*, *CASP8*, and *CASP9* in the apoptosis signaling pathway (Figure 1E,G). In summary, pyruvate promoted GC proliferation but inhibited GC apoptosis of follicles to accelerate the pubertal initiation in mice.

### 3.2. Pyruvate Promotes the Proliferation and Inhibits the Apoptosis of GCs

**Figure 2:**
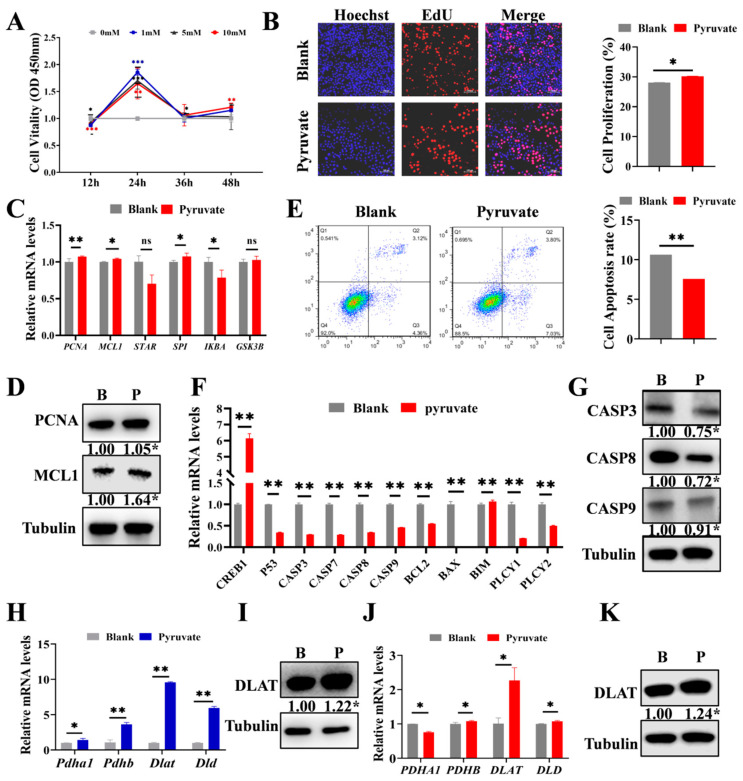
Pyruvate promotes the proliferation but inhibits the apoptosis of GCs. (**A**). Cell viability of GCs exposed to pyruvate were measured by the CCK-8 assay. (**B**)**.** EdU detection of proliferation rate after pyruvate treatment. Scale bar: 200 μm. The proliferation signaling pathway genes’ mRNA (**C**) and protein expressions (**D**) in GCs in the blank group (B) and the pyruvate group (P). (**E**). Flow cytometry assay of the apoptosis rate after pyruvate treatment. The apoptotic signaling pathway genes’ mRNA (**F**) and protein expressions (**G**) in GCs in the blank group (B) and the pyruvate group (P). (**H**). The mRNA levels of pyruvate dehydrogenase complex in mice ovaries. (**I**). The protein level of DLAT in mice ovaries. (**J**). The mRNA levels of pyruvate dehydrogenase complex in GCs. (**K**). The protein level of DLAT in GCs. ^ns^ *p* > 0.05, * *p* < 0.05, ** *p* < 0.01 and *** *p* < 0.001.

GCs are intimately associated with follicular growth [6,32]. Therefore, we further validated the function of pyruvate on GCs. The cells were treated with four concentrations (0 mM, 1 mM, 5 mM, and 10 mM) of pyruvate, and then the cell viability was assayed at 12 h, 24 h, 36 h, and 48 h of treatment. Based on the results, 1 mM for 24 h demonstrated optimal cell viability and was selected for the follow-up experiments (Figure 2A). Pyruvate significantly promoted GC proliferation (Figure 2B). Furthermore, the mRNA levels of *PCNA*, *MCL1*, *STAR*, *SP1*, *IKBA*, and *GSK3B* of the cell proliferation signaling pathway, as well as the protein levels of PCNA and MCL1, were significantly up-regulated after pyruvate treatment (Figure 2C,D). Flow cytometry showed that pyruvate significantly inhibited GC apoptosis (Figure 2E). Pyruvate decreased the mRNA levels of *CREB1*, *P53*, *CASP3*, *CASP7*, *CASP9*, *BCL-2*, *BIM*, *PLCY1*, and *PLCY2* of the apoptosis pathway (Figure 2F). Additionally, pyruvate treatment markedly reduced the protein expressions of CASP3, CASP8, and CASP9 (Figure 2G). In addition, we found that pyruvate significantly up-regulated the mRNA and protein levels of *DLAT*, a subunit of the pyruvate dehydrogenase complex [33,34], in both ovaries (Figure 2H,I) and GCs (Figure 2J,K).

### 3.3. DLAT Accelerates GC Proliferation While Attenuating Apoptosis

**Figure 3:**
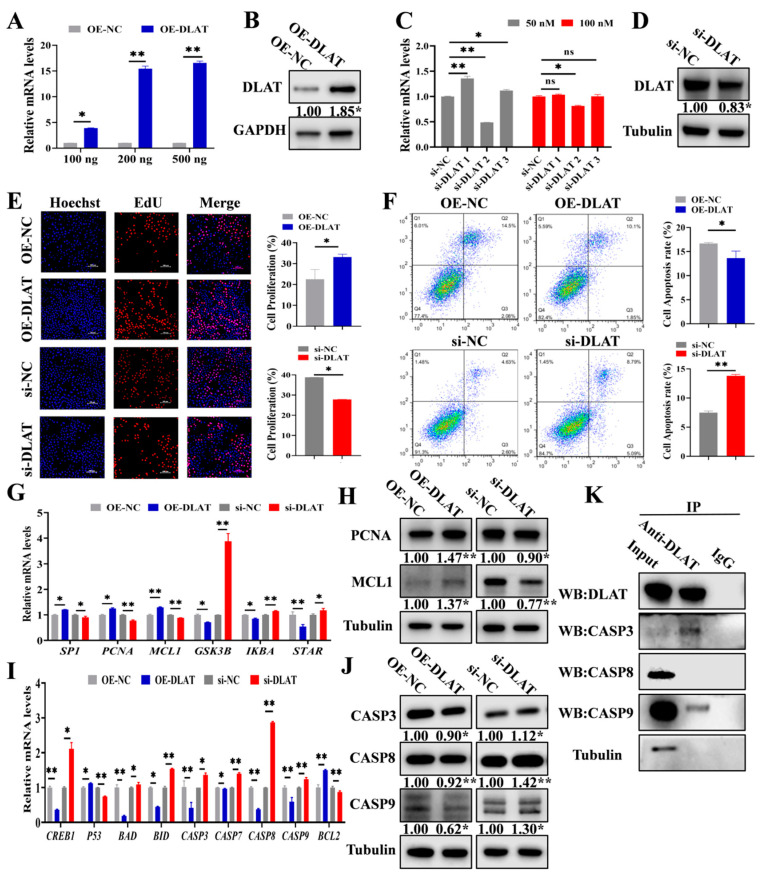
*DLAT* accelerates GC proliferation while attenuating apoptosis. (**A**). The mRNA expressions of *DLAT* after transfection with 100 ng, 200 ng, and 500 ng of OE-DLAT. (**B**). The protein level of DLAT after transfection with 200 ng of OE-DLAT. (**C**). The mRNA levels of *DLAT* after transfection with 50 nM and 100 nM of si-DLATs (si-DLAT1, si-DLAT2, and si-DLAT3). (**D**). The protein level of DLAT after transfection with 50 nM of si-DLAT2. (**E**). EdU detection of proliferation rate transfected with OE-DLAT or si-DLAT. Scale bar: 200 μm. (**F**). Apoptosis rates were assessed by flow cytometry in cells transfected with OE-DLAT or si-DLAT. The mRNA levels (**G**) and protein (**H**) expressions of proliferation signaling pathways, along with the mRNA levels (**I**) and protein expressions (**J**) of apoptotic signaling pathways in GCs transfected with OE-DLAT or si-DLAT. (**K**). Co-immunoprecipitation (Co-IP) results of *DLAT* antibody pull-down of other proteins in GCs. ^ns^ *p* > 0.05, * *p* < 0.05, and ** *p* < 0.01.

To investigate the function of *DLAT* on GCs, the *DLAT* overexpression vector (OE-DLAT) and three *DLAT* small interfering RNA (si-DLAT1, si-DLAT2, and si-DLAT3) were synthesized. A total of 100 ng, 200 ng, and 500 ng of OE-DLAT and 50 nM and 100 nM of *DLAT* siRNAs were transfected into GCs for confirming the efficiency of *DLAT* overexpression and knockdown. A total of 200 ng of OE-DLAT (Figure 3A,B) and 50 nM of si-DLAT2 (si-DLAT) (Figure 3C,D) demonstrated optimal efficiency and were selected for the subsequent experiments. OE-DLAT significantly promoted the proliferation of GCs, while si-DLAT significantly inhibited the proliferation of GCs (Figure 3E). Flow cytometry showed that OE-DLAT significantly decreased the apoptosis rates in the GCs, but si-DLAT exhibited the opposite effects (Figure 3F). OE-DLAT significantly elevated the mRNA and protein levels of *PCNA* and *MCL1* (Figure 3G), whereas si-DLAT exhibited the contrary effects (Figure 3H). OE-DLAT significantly inhibited the mRNA and protein expressions of *CASP3*, *CASP8*, and *CASP9*, while si-DLAT had the opposite effects (Figure 3I,J). Notably, we found that DLAT protein interacted with the apoptosis-related proteins CASP3 and CASP9 (Figure 3K).

### 3.4. DLAT Promotes the Growth of Porcine Follicles

**Figure 4:**
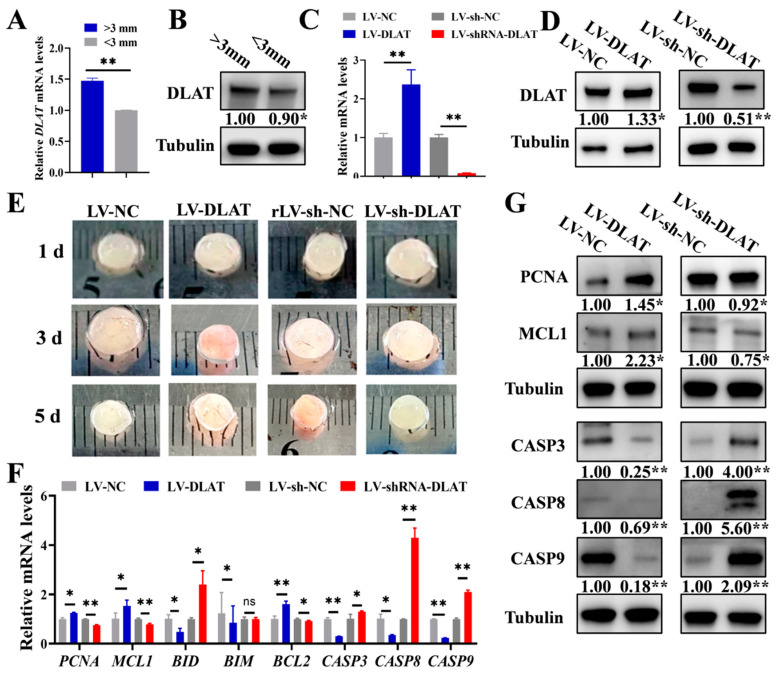
*DLAT* supports the growth of follicles in pigs. The expression levels of *DLAT* mRNA (**A**) and protein (**B**) in large follicles (>3 mm) versus small follicles (<3 mm). *DLAT* mRNA levels (**C**) and protein expression (**D**) in follicles treated with LV-DLAT or LV-sh-DLAT. (**E**). The appearance of porcine follicles on the first, third, and fifth day after transfection with LV-DLAT or LV-sh-DLAT. The mRNA (**F**) and protein levels (**G**) of the proliferation and apoptotic signaling pathway in porcine follicles after transfection with LV-DLAT or LV-sh-DLAT. ^ns^ *p* > 0.05, * *p* < 0.05, and ** *p* < 0.01.

Based on the above results, *DLAT* expression was analyzed in different follicles, which showed that large follicles (>3 mm) displayed significantly greater mRNA and protein levels of *DLAT* compared to small follicles (<3 mm) (Figure 4A,B). It indicated that with the growth of follicles, the expression of *DLAT* was gradually up-regulated. To analyze the contribution of *DLAT* to follicular growth, the lentiviral vector overexpression of *DLAT* (LV-DLAT) or knockdown of *DLAT* (LV-sh-DLAT) was infected into porcine follicles in vitro. LV-DLAT markedly elevated the mRNA and protein expressions of *DLAT* (Figure 4C,D) and stimulated blood vessel formation in follicles as well as decreased the turbidity of the follicular fluid in pigs, while LV-sh-DLAT had the opposite results (Figure 4E). Several investigations have revealed that blood vessels within follicles are vital for their growth and development [24,25]. Subsequently, we assessed the expression levels of the proliferation and apoptosis signaling pathways in follicles. LV-DLAT significantly increased the mRNA (Figure 4F) and protein (Figure 4G) levels of *PCNA* and *MCL1*, while LV-sh-DLAT showed the opposite results. In addition, LV-DLAT significantly decreased the mRNA (Figure 4F) and protein (Figure 4G) levels of *CASP3*, *CASP8,* and *CASP9*, while LV-sh-DLAT resulted in the opposite effects. Overall, *DLAT* is essential for follicular growth by enhancing vascularization and GC proliferation while also suppressing GC apoptosis.

### 3.5. DLAT Stimulates Follicular Growth in Mice

**Figure 5:**
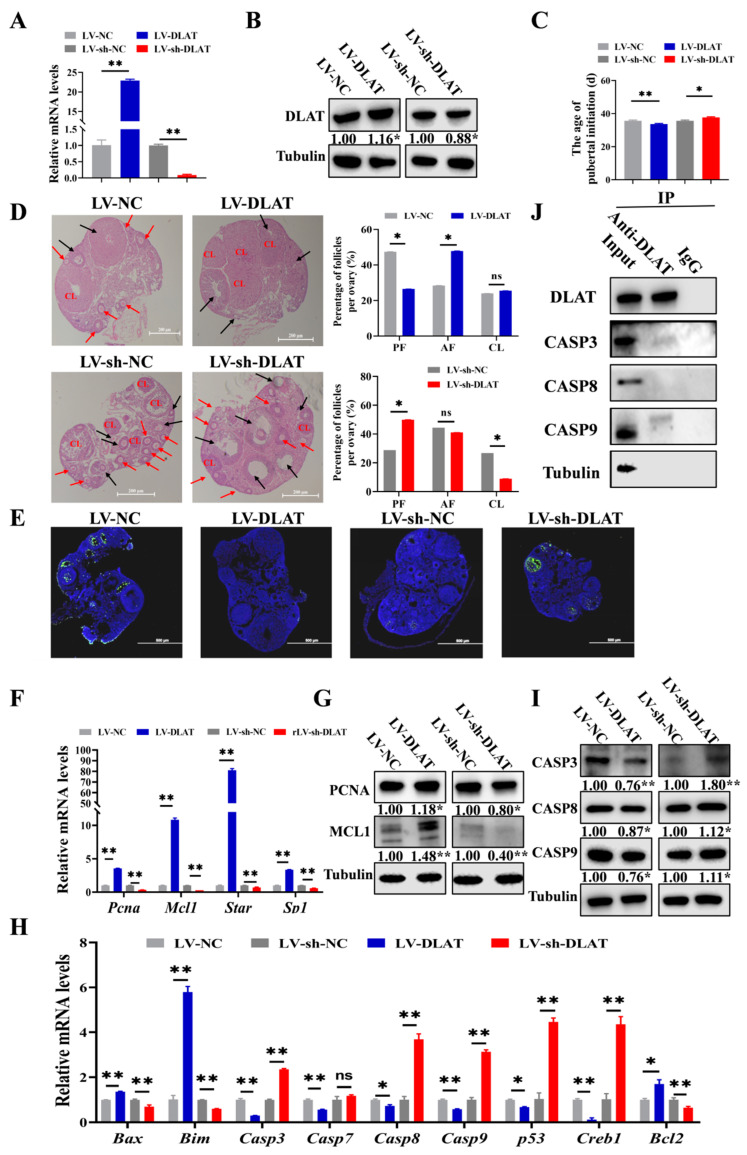
DLAT stimulates follicular growth in mice. The mRNA (**A**) and protein level (**B**) of *DLAT* in mice ovaries treated with LV-DLAT or LV-sh-DLAT. (**C**). The age of pubertal initiation in mice treated with LV-DLAT or LV-sh-DLAT. (**D**). The results of HE staining in mice ovaries treated with LV-DLAT or LV-sh-DLAT. Black arrows: PF, red arrows: AF, and CL: corpus luteum. Scale bar: 500 μm (**E**). The results of TUNEL in mice ovaries treated with LV-DLAT or LV-sh-DLAT, and the green fluorescence represented apoptotic cells. Scale bar: 200 μm. The mRNA levels (**F**) and protein expression (**G**) of the proliferation signaling pathway in mice treated with LV-DLAT or LV-sh-DLAT. The mRNA levels (**H**) and protein expression (**I**) of the apoptosis signaling pathway in mice ovaries treated with LV-DLAT or LV-sh-DLAT. (**J**). Co-IP results of DLAT antibody pull-down of other proteins in mice ovaries. ^ns^ *p* > 0.05, * *p* < 0.05, and ** *p* < 0.01.

In previous experiments, we validated the function of *DLAT* in GCs and follicles in vitro. Next, we explored its role in follicular growth in vivo. Three-week-old mice received intraperitoneal injections of lentivirus, and the efficiency assessment was performed by detecting *DLAT* mRNA and protein expression through RT-qPCR and Western blot (Figure 5A,B). Compared to LV-NC, LV-DLAT significantly advanced the age of pubertal initiation, whereas LV-sh-DLAT, relative to LV-sh-NC, markedly delayed the age of pubertal initiation (Figure 5C). Additionally, LV-DLAT significantly decreased the proportion of PFs but significantly increased the proportion of AFs. LV-sh-DLAT significantly increased the proportion of PFs while decreasing the proportion of CL (Figure 5D), which indicated that *DLAT* promoted follicular growth. Furthermore, TUNEL showed that LV-DLAT inhibited the apoptosis of GCs, while LV-sh-DLAT promoted the apoptosis of GCs (Figure 5E). The mRNA and protein levels of the proliferation (Figure 5F,G) and apoptosis (Figure 5H,I) signaling pathway further indicated that *DLAT* promoted GC proliferation and follicular growth. Similarly, we also found that DLAT protein bound with CASP3 and CASP9 proteins (Figure 5J), which was consistent with previous results (Figure 3K). In conclusion, DLAT interacted with CASP3 and CASP9 proteins to inhibit the apoptosis of GCs, and promoted follicular growth, thereby advancing the age of pubertal initiation of mice.

## 4. Discussion

Pyruvate serves as a key precursor in the TCA cycle that is necessary for cellular growth processes [35,36]. Follicular growth is accompanied by increasing energy expenditure, and studies have shown that pyruvate levels in oocytes increase 2-fold and 9-fold, respectively, during the primary and pre-ovulation stages of the follicles [37,38]. Conversely, decreased pyruvate leads to energy deficiency, which in turn results in GC apoptosis [39] and inhibits follicular growth [40,41]. In our study, we found that pyruvate significantly increased the proportion of AFs (Figure 1B) and advanced the age of pubertal initiation in mice (Figure 1A). This suggests that pyruvate may play a crucial role in regulating the onset of puberty. An excessive intake of pyruvate might lead to an earlier age at puberty, which might be detrimental to normal body development. Conversely, pyruvate supplementation can be used to ameliorate the delay in age at puberty. Previous studies have shown that GCs promote the activation of primordial follicles by enhancing glycolysis [37,42]. These findings suggest a positive role for pyruvate in follicular growth. Moreover, pyruvate increased the expression levels of the proliferation signaling pathway such as *PCNA* to promote GC proliferation (Figure 2B) while decreasing the expression levels of the apoptosis signaling pathway such as *CASP3* to inhibit GC apoptosis (Figure 2E–G). These appearances were consistent with another study in which pyruvate inhibited apoptosis in other cells [43]. Although the 2–3% difference in apoptosis between the pyruvate and control groups appeared to be minor, studies have reported small changes in apoptosis rates [44,45], and follow-up experiments have showed that a minor difference significantly improved the ovarian reserve and fertility of mice. Thus, pyruvate promotes follicular growth by promoting GC proliferation and inhibiting GC apoptosis.

To explore the mechanism by which pyruvate promotes follicular growth, we examined the expression of the *PDHA1*, *PDHB*, *DLAT*, and *DLD* of pyruvate dehydrogenase complex after pyruvate treatment and found that the mRNA levels of all these genes were significantly up-regulated (Figure 2H,J), with *DLAT* showing the most significant increase. It is well known that *DLAT* is an important enzyme in the pyruvate metabolic pathway, catalyzing the transfer of acetyl groups to CoA, and studies have shown that pyruvate, as a precursor of acetyl-CoA, may regulate *DLAT* expression through histone acetylation [46]. Furthermore, ATP produced from pyruvate metabolism activates AMPK [47], which may influence *DLAT* expression. Additionally, we observed higher expression levels of *DLAT* in large follicles compared with small follicles (Figure 4A,B). Follicular growth is accompanied by an increase in volume [48], and the follicular size is closely related to the follicular growth of and the meiotic competence of oocytes [49]. In cows, follicles > 6 mm have about twice the developmental potential of smaller antral follicles [50]. The results indicate that pyruvate promotes follicular growth by up-regulating the expression of *DLAT.*

We also found that *DLAT* promoted GC proliferation (Figure 3E) and inhibited GC apoptosis (Figure 3F). The results were consistent with our previous works (Figure 2B,E), indicating that pyruvate up-regulated the expression of *DLAT* to promote GC growth. After LV-DLAT infection, the follicle surface blood vessels were prominent and pink in color. However, after LV-sh-DLAT infection, the follicle surface had almost no blood vessels and appeared white (Figure 4E). These results showed that *DLAT* promoted the formation of follicular blood vessels and decreased the turbidity of the follicular fluid in pigs. The growth of follicles is accompanied by increased vascularization [51,52]. Researchers found that inhibiting vascularization by antagonizing the HIF-1α-VEGF axis in GCs compromises follicle viability and leads to defective follicular growth [53]. Thus, *DLAT* promoted follicular growth by enhancing vascularization. Furthermore, LV-DLAT inhibited GC apoptosis (Figure 5E), significantly doubled the number of antral follicles (Figure 5D), and advanced pubertal initiation (Figure 5C) in mice, which was in line with pyruvate treatment. These results suggest that *DLAT* promotes follicular growth by enhancing vascularization, promoting GC proliferation, and inhibiting GC apoptosis.

Based on the result that *DLAT* inhibited GC apoptosis, we investigated its role in apoptosis. The caspase family is important in the activation and execution of apoptotic signals [54], regulating cell apoptosis primarily through membrane receptor pathways, endoplasmic reticulum pathways, and mitochondrial pathways [55,56,57]. Given that *DLAT* is localized in mitochondria, we focused on mitochondrial pathways, where CASP9 combined with Apaf-1 to trigger the apoptotic signal and then activating CASP3 and inducing cell apoptosis [58]. In this study, Co-IP validated the interaction between DLAT protein and CASP3 and CASP9 proteins in both GCs (Figure 3K) and mice (Figure 5J). In a word, these results enhance our understanding of the follicular growth process and provide new insights and therapeutic directions for addressing disorders of the female reproductive system.

In this study, we have conducted validations of the functions of pyruvate and *DLAT* in humans, mice, and pigs, and the results showed broad applicability and reliability. However, the experimental results on porcine follicles require further validation because the *DLAT* antibody we used is suitable for humans and mice. Pyruvate promoted the development of GCs, the maturation of oocytes, and ovulation [21]. Therefore, pyruvate holds considerable potential in the treatment of infertility. Although the KGN line is a cancerous cell, it retains some key functions of normal GCs, making it a widely used model cell line in numerous studies [59,60]. However, it must be acknowledged that there are certain differences between KGN cells and healthy physiological models. In addition, the knockdown efficiency of DLAT protein was only 17%, and this may affect the accuracy of experimental results. The findings will be more credible if the knockdown efficiency is improved using CRISPR-Cas9. These are the limitations and shortcomings we face in our research.

## 5. Conclusions

In summary, our current findings reveal that the pyruvate could increase the mRNA and protein levels of *DLAT* and promote the expression of proliferation signaling pathway genes to promote GC proliferation. Moreover, DLAT bound to CASP3 and CASP9 proteins, and inhibited the apoptosis of GCs to promote follicular development, ultimately advancing the age of pubertal initiation of mice.

## Figures and Tables

**Table 1:** Primers used in mouse.

Gene Name	Primer Sequences (5′ → 3′)
*Bcl2* (mouse)	F: CCTGTGGATGACTGAGTACCTG
	R: AGCCAGGAGAAATCAAACAGAGG
*Casp3* (mouse)	F: GGAGTCTGACTGGAAAGCCGAA
	R: CTTCTGGCAAGCCATCTCCTCA
*Casp7* (mouse)	F: CCGTCCACAATGACTGCTCTTG
	R: CCCGTAAATCAGGTCCTCTTCC
*Casp8* (mouse)	F: GCTGACTTTCTGCTGGGGAT
	R: GACATCGCTCTCTCAGGCTC
*Casp9* (mouse)	F: GCTGTGTCAAGTTTGCCTACCC
	R: CCAGAATGCCATCCAAGGTCTC
*p53* (mouse)	F: GGCAGACTTTTCGCCACAG
	R: GATGATGGTAAGGATAGGTCGG
*Bim* (mouse)	F: GGAGATACGGATTGCACAGGAG
	R: CTCCATACCAGACGGAAGATAAAG
*Creb1* (mouse)	F: CACAGACCACTGATGGACAGCA
	R: AGGACGCCATAACAACTCCAGG
*Bax* (mouse)	F: AGGATGCGTCCACCAAGAAGCT
	R: TCCGTGTCCACGTCAGCAATCA
*Pcna* (mouse)	F: CAAGTGGAGAGCTTGGCAATGG
	R: GCAAACGTTAGGTGAACAGGCTC
*Mcl1* (mouse)	F: AGCTTCATCGAACCATTAGCAGAA
	R: CCTTCTAGGTCCTGTACGTGGA
*Star* (mouse)	F: GTGCTTCATCCACTGGCTGGAA
	R: GTCTGCGATAGGACCTGGTTGA
*Sp1* (mouse)	F: CTCCAGACCATTAACCTCAGTGC
	R: CACCACCAGATCCATGAAGACC
*Gapdh* (mouse)	F: GGACTCATGACCACGGTCCAT
	R: TCAGATCCACAACCGACACGT
*Dlat* (mouse)	F: CTGAGTGAAGGAGACTTGCTGG
	R: TCCCAGAGGAACATCCCTTGTG

**Table 2:** Primers used in human.

Gene Name	Primer Sequences (5′ → 3′)
*BCL2* (human)	F: ATCGCCCTGTGGATGACTGAGT
	R: GCCAGGAGAAATCAAACAGAGGC
*CASP3* (human)	F: GGAAGCGAATCAATGGACTCTGG
	R: GCATCGACATCTGTACCAGACC
*CASP7* (human)	F: CGGAACAGACAAAGATGCCGAG
	R: AGGCGGCATTTGTATGGTCCTC
*CASP8* (human)	F: GCTGACTTTCTGCTGGGGAT
	R: GACATCGCTCTCTCAGGCTC
*CASP9* (human)	F: GTTTGAGGACCTTCGACCAGCT
	R: CAACGTACCAGGAGCCACTCTT
*P53* (human)	F: CCTCAGCATCTTATCCGAGTGG
	R: TGGATGGTGGTACAGTCAGAGC
*BIM* (human)	F: CAAGAGTTGCGGCGTATTGGAG
	R: ACACCAGGCGGACAATGTAACG
*CREB1* (human)	F: GACCACTGATGGACAGCAGATC
	R: GAGGATGCCATAACAACTCCAGG
*BAX* (human)	F: TCAGGATGCGTCCACCAAGAAG
	R: TGTGTCCACGGCGGCAATCATC
*IKBA* (human)	F: TCCACTCCATCCTGAAGGCTAC
	R: CAAGGACACCAAAAGCTCCACG
*GSK3B* (human)	F: CCGACTAACACCACTGGAAGCT
	R: AGGATGGTAGCCAGAGGTGGAT
*PCNA* (human)	F: CAAGTAATGTCGATAAAGAGGAGG
	R: GTGTCACCGTTGAAGAGAGTGG
*MCL1* (human)	F: CCAAGAAAGCTGCATCGAACCAT
	R: CAGCACATTCCTGATGCCACCT
*STAR* (human)	F: TACGTGGCTACTCAGCATCGAC
	R: TCAACACCTGGCTTCAGAGGCA
*SP1* (human)	F: ACGCTTCACACGTTCGGATGAG
	R: TGACAGGTGGTCACTCCTCATG
*GAPDH* (human)	F: TGTTCGTCATGGGTGTGAAC
	R: ATGGCATGGACTGTGGTCAT
*DLAT* (human)	F: TTGATGTCAGTGTTGCGGTCAGTAC
	R: GTGGCTGTAGTTTACCCTCTCTTGC

**Table 3:** Primers used in pig.

Gene Name	Primer Sequences (5′ → 3′)
*BCL2* (pig)	F: GAGTTCGGTGGGGTCATGTG
	R: TACAGCTCCACAAAGGCATCC
*CASP3* (pig)	F: AAGAAAACAGCATTACCCTCCTTG
	R: TTCAGAGGGGACTGCTGTAGA
*CASP8* (pig)	F: CTCTGCCTACAGGGTCATGC
	R: AGGATGGCCCTCTTCTCCAT
*CASP9* (pig)	F: AACTTCTGCCATGAGTCGGG
	R: CTGGCCTTGGCAGTCAGG
*BIM* (pig)	F: ATCCTCCCTGCTGTCTCGAT
	R: AAGAAAACAGCATTACCCTCCTTG
*BID* (pig)	F: ACGAGCGCATCACAAACCTA
	R: GCCTCCTGGCTCTCAGAATC
*PCNA* (pig)	F: AAGAGGAGGAAGCAGTTACCA
	R: TCATCTTCGATCTTGGGAGCC
*MCL1* (pig)	F: GGAAGGCGTTAGAGACCCTG
	R: GTCACAATCCTGCCCCAGTT
*GAPDH* (pig)	F: GGACTCATGACCACGGTCCAT
	R: TCAGATCCACAACCGACACGT
*DLAT* (pig)	F: AGCAGCACGACTAGAGGGTATGG
	R: TACCAGCGGCGGTTAGGAGAC

**Table 4:** Primers used for vector construction.

Gene Name	Primer Sequences (5′ → 3′)
*DLAT*	F: **GGATCC**CCCGCATCAGAAGGTTCCAT
	R: **GAATTC**TCAGTGTGACCTGGGAGAGT

## Data Availability

The data are contained within this article.
